# Storage conditions of intestinal microbiota matter in metagenomic analysis

**DOI:** 10.1186/1471-2180-12-158

**Published:** 2012-07-30

**Authors:** Silvia Cardona, Anat Eck, Montserrat Cassellas, Milagros Gallart, Carmen Alastrue, Joel Dore, Fernando Azpiroz, Joaquim Roca, Francisco Guarner, Chaysavanh Manichanh

**Affiliations:** 1 Digestive System Research Unit, Vall d’Hebron Institut de Recerca, Ciberehd, 08035, Barcelona, Spain; 2 Institut National de Recherche Agronomique, Micalis UMR1319, Domaine de Vilvert, 78352, Jouy-en-Josas, France; 3 Molecular Biology Institute of Barcelona, IBMB-CSIC, 08028, Barcelona, Spain

**Keywords:** Needs for standardization/RNA and DNA degradation/Metagenomics/16S ribosomal RNA

## Abstract

**Background:**

The structure and function of human gut microbiota is currently inferred from metagenomic and metatranscriptomic analyses. Recovery of intact DNA and RNA is therefore a critical step in these studies. Here, we evaluated how different storage conditions of fecal samples affect the quality of extracted nucleic acids and the stability of their microbial communities.

**Results:**

We assessed the quality of genomic DNA and total RNA by microcapillary electrophoresis and analyzed the bacterial community structure by pyrosequencing the 16S rRNA gene. DNA and RNA started to fragment when samples were kept at room temperature for more than 24 h. The use of RNAse inhibitors diminished RNA degradation but this protection was not consistent among individuals. DNA and RNA degradation also occurred when frozen samples were defrosted for a short period (1 h) before nucleic acid extraction. The same conditions that affected DNA and RNA integrity also altered the relative abundance of most taxa in the bacterial community analysis. In this case, intra-individual variability of microbial diversity was larger than inter-individual one.

**Conclusions:**

Though this preliminary work explored a very limited number of parameters, the results suggest that storage conditions of fecal samples affect the integrity of DNA and RNA and the composition of their microbial community. For optimal preservation, stool samples should be kept at room temperature and brought at the laboratory within 24 h after collection or be stored immediately at −20°C in a home freezer and transported afterwards in a freezer pack to ensure that they do not defrost at any time. Mixing the samples with RNAse inhibitors outside the laboratory is not recommended since proper homogenization of the stool is difficult to monitor.

## Background

The human gut microbiome is a highly dense microbial ecosystem, largely outnumbering our own eukaryotic body cells. Its intimate contact with our digestive system and its potential role in health and disease states makes this ecosystem very attractive for a deep characterization of its composition and function. In recent years, high-throughput sequencing has been the catalyst for analyzing microbial population diversity and functions. While bacterial 16S rRNA gene survey can answer the question “which species are there” [1], functional metagenomics can also address “what are they doing” by examining the sequences of genomic fragments and by exploiting, for instance, gene expression analysis by metatranscriptomics [2-4]. These approaches allow not only the characterization of individual organisms and their genes; but also metabolic and regulatory pathways, functional interactions inside a microbial community and crosstalk between a microbial community and its host.

Functional metagenomic projects are highly interdisciplinary and involve numerous procedures, ranging from clinical protocols for sample collection to bioinformatics tools for data interpretation. Strong biases can be introduced in each of these steps. Sample storage conditions, one of the first steps, is critical for downstream analyses. Previous studies had indicated that storing conditions of stool samples only modestly affect the structure of their microbial community [5-8]. However, little is known about the influence of storing conditions on more deep structural and functional analyses, which require maximal integrity of genomic DNA and RNA. Intact DNA fragments are critical for metagenomic library construction [9-11] and to characterizing intact genetic pathways either by sequence-based or function screening-based approaches [12,13]. Moreover, excessive degradation of DNA reduces the efficiency of shotgun sequencing [2]. The recovery of total RNA with high integrity is necessary for proper cDNA synthesis and absolutely essential for describing the gene expression in a community sample [4,14-16].

In the present study, we compared the effect of different storage conditions of stool samples on microbial community composition, genomic DNA and total RNA integrity.

## Results and discussion

### Effect of storage conditions on genomic DNA

In order to investigate the effect of storage conditions on the quality of genomic DNA, we chose a subset of stool samples collected by 4 volunteers (#1, #2, #3 and #4) and that had been stored in the following 6 conditions: immediately frozen at −20°C (F); immediately frozen (UF) and then unfrozen during 1 h and 3 h; kept at room temperature (RT) during 3 h, 24 h and 2 weeks. In this case, all 24 samples were kept at −80°C in the laboratory until genomic DNA was extracted and its integrity analyzed using microcapillary electrophoresis.

**Figure 1:**
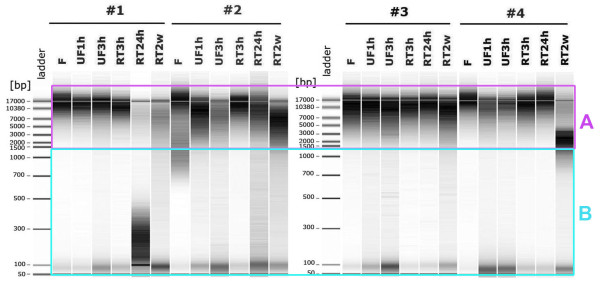
**Fragmentation analysis of genomic DNA.** Microcapillary electrophoresis patterns of genomic DNA extracted from fecal samples collected by 4 individuals (#1, #2, #3, #4) and stored in the following conditions: immediately frozen at −20°C (F); immediately frozen and then unfrozen during 1 h and 3 h (UF1h, UF3h); kept at room temperature during 3 h, 24 h and 2 weeks (RT3h, RT24h, RT2w). The equivalent to 1 mg of fecal material is loaded on each lane. A DNA fragment size (base pair) ladder was loaded in the left most lanes.

In all the tested conditions the amount of DNA obtained was in the range of 70–235 μg/250 mg of fecal sample, which is sufficient for downstream analysis such as metagenomic library construction or shotgun sequencing [2]. As illustrated in figure 1 microcapillary electrophoresis revealed that genomic DNA was mostly preserved as high-molecular weight fragments when samples were stored immediately after collection at −20°C in a home freezer or left up to 3 h at room temperature. However, DNA became fragmented when samples were allowed to unfreeze during 1 h (subjects #2 and #3) or stored at room temperature over 24 h (subjects #1 and #2). DNA degradation further increased and nearly all high-molecular weight fragments disappeared when samples had been kept over 2 weeks at room temperature (#1, #2 and #3). In order to provide a semi-quantitative comparison, we extracted the signal intensity from the gel using the ImageJ software. This signal is converted into a number that is proportional to the DNA quantity. As shown in figure 1, we used the upper size-range (rectangle A) of the frozen sample as a proxy for “no degraded DNA” and the lower size-range (rectangle B) for “degraded DNA” (figure 1). The threshold of 1.5 kb was used to discriminate the 2 size-ranges, since it is recommended for shotgun sequencing in the 454 protocol from Roche Applied Science. Proportion of degraded DNA for each sample was then calculated by the ratio between the lower size-range intensity and the total intensity. Our results, displayed in Table 1, showed a significant degradation (*p* < 0.01, Poisson regression analysis) for all storage conditions compared to frozen samples except those kept at room temperature for 3 h. Therefore, storing fecal samples at room temperature over 3 h after collection or allowing them to thaw and refreeze is not recommended for shotgun metagenomic sequencing, since DNA extracted from these samples can be significantly fragmented. 

**Table 1:** Percentage of DNA compared to the frozen samples

	**% degraded DNA**				**n = 4**
	**#1**	**#2**	**#3**	**#4**	***p*value when compared to frozen samples**
F	12	28	10	9	
UF1h	12	24	23	34	< 0.01
UF3h	25	39	31	34	< 0.001
RT3h	17	16	12	15	0.9270
RT24h	84	44	13	15	< 0.001
RT2w	48	38	26	40	< 0.001

Statistical analysis was performed using Poisson regression model; *p* value < 0.05 is considered significant; #1, #2, #3, #4 correspond to subjects 1, 2, 3, 4; F = frozen; UF1h = unfrozen during 1 h; UF3h = unfrozen during 3 h; RT = room temperature; 2w = 2 weeks.

Even though mechanical disruption of the samples used in our extraction method could damage the integrity of large DNA molecules, we believe that storage conditions, more than directly degrade DNA during storage period or the extraction step, dysregulate cellular compartments and activate enzymatic activities (i.e. nucleases). Further studies could be designed in order to test the effect of different extraction methods including mechanical or non-mechanical disruption on DNA integrity.

### Effect of storage conditions on microbial diversity

Although storage conditions of stool samples greatly affected the integrity of bacterial DNA, this observation did not demonstrate an impediment for metagenomic analyses. In order to verify this extreme, we examined to which extent storage conditions could bias intestinal microbial composition. By using the genomic DNA extracted from the 24 samples obtained from the 4 above cited volunteers (#1, #2, #3 and #4), we PCR-amplified the V4 region of the 16S rRNA gene and sequenced the products using a GS FLX 454 pyrosequencer. We obtained a total of 127,275 high quality sequences, which we then analyzed using the Qiime pipeline to determine and compare the microbial diversity.

We validated the presence of a bacterial species or taxon when its abundance was higher than 0.2% in at least one sample. Accordingly, we identified a total of 188 taxa after validating an average of 3,400 sequences and 114 taxa per sample (see Additional file 1: Table S1). These 188 species classified into 48 genera and 4 phyla as follows: Firmicutes (48%), Bacteroidetes (46%), Actinobacteria (5%) and Proteobacteria (1%).

**Figure 2:**
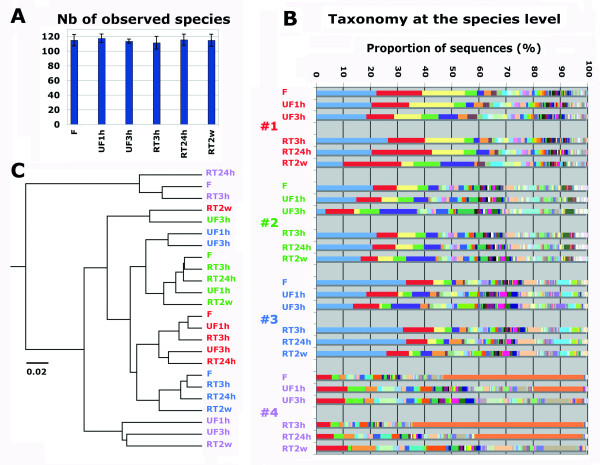
**Bacterial community analysis based on 16S rRNA gene survey.A**) Alpha-diversity analysis of number of species observed in 6 storage conditions: Immediately frozen (F); unfrozen 1 h and 3 h (UF1h, UF3h); room temperature 3 h, 24 h, and 2 weeks (RT3h, RT24h, RT2w). The plot averages the number of species from the samples provided by 4 individuals in each condition. **B**) Taxonomy analysis at the species level of the 24 samples based on alignment performed using PyNast against Silva 108 release database and OTUs assignment using blast and the Silva 108 release taxa mapping file. Individual #1 (red), #2 (blue), #3 (green), #4 (purple). A more detailed taxonomy assignment is provided in the additional data (See Additional file 3: Table S1). **C**) UPGMA clustering of the 24 samples based on weighted UniFrac method. Samples from the 4 individuals are colored as in B. The scale bar represents 2% sequence divergence.

Alpha-diversity analysis showed that the storage procedures did not influence the total number of observed taxa (figure 2A) and did not greatly alter the bacterial composition of the samples at the phylum level (See Additional file 2: Figure S1) except the samples from subject #4. However, the storage conditions had a large impact on the taxonomic composition of the samples at the genus and species level for all subjects (figure 2B). Variations were found depending on both the storage condition and the individual. In Table 2, we showed the effect of storage conditions on the proportion of 3 main bacterial taxa. As shown in this table, the abundance comparison between frozen and unfrozen samples was affected by thawing samples for 1 h and 3 h as exemplified by the significant decrease of a dominant unknown taxon from the *Bacteroides* genus (from an average of 19% (F) to 13% (UF1h; *p* = 0.044, Poisson regression model) and to 9% (UF3h; *p* < 0.0001, Poisson regression model)). The proportion of the two other bacterial taxa was significantly affected when thawing the samples over 3 h (*p* = 0.02 and *p* = 0.0007 respectively, Poisson regression model). The room temperature condition was only significantly affecting the bacterial proportion after 2 weeks (*p* < 0.04 for all taxa, Poisson regression model) as shown in Table 3.

**Table 2:** Taxonomic comparison for 3 main bacterial taxa between frozen and unfrozen samples

**Taxon**	F*	UF1h*	UF3h*	***p *value F vs UF1h**	***p *value F vs UF3h**
*Bacteroides*;uncultured bacterium	19	13	9	0.044	9.68e-05
Prevotellaceae;uncultured;human gut metagenome	7	6	3	0.6804	0.0222
*Bifidobacterium*;uncultured bacterium	2	4	8	0.2257	0.0007

Statistical analysis was performed using Poisson regression model; *p* value < 0.05 is considered significant; n = 4 subjects; * Values are mean proportion of sequences (%).

F = frozen; UF1h = unfrozen during 1 h; UF3h = unfrozen during 3 h; Taxonomy is indicated at the genus level and if not possible at the family level.

**Table 3:** Taxonomic comparison for 3 main bacterial taxa between frozen and RT samples

**Taxon**	F*	RT3h*	RT24h*	RT2w*	***p *value F vs RT3h**	***p *value F vs RT24h**	***p *value F vs RT2w**
*Bacteroides*;uncultured bacterium	19	20	19	13	0.749	0.749	0.0349
Prevotellaceae;uncultured;human gut metagenome	7	6	5	3	0.6804	0.3189	0.0140
*Bifidobacterium*;uncultured bacterium	2	2	3	7	1	0.3964	0.0030

Statistical analysis was performed using Poisson regression model. * Values are mean proportion of sequences (%). *p*-value < 0.05 is considered significant; n = 4 subjects; F = frozen; UF1h = unfrozen during 1 h; UF3h = unfrozen during 3 h; RT = room temperature; 2w = 2 weeks; Taxonomy is indicated at the genus level and if not possible at the family level.

To further compare the 24 samples, we used the weighted Unifrac UPGMA method to build a clustering tree. The result showed that frozen samples, 3 h and 24 h room temperature samples tend to cluster together and far from the defrosted and 2 weeks room temperature samples (figure 2C). This analysis also indicated that, under these later conditions, intra-individual variability became higher than inter-individual one.

The above analyses on the effect of storage conditions on microbial diversity corroborate previous observations showing a relative stable community composition when stool samples are kept up to 24 h at room temperature [8]. However, our study reveals that under more prolonged conditions (i.e. 2 weeks room temperature) or by changing temperature (i.e. unfreezing samples during only 1 or 3 h), the relative abundances of most taxa can be greatly altered in the bacterial community.

### Effect of storage conditions on total RNA

**Figure 3:**
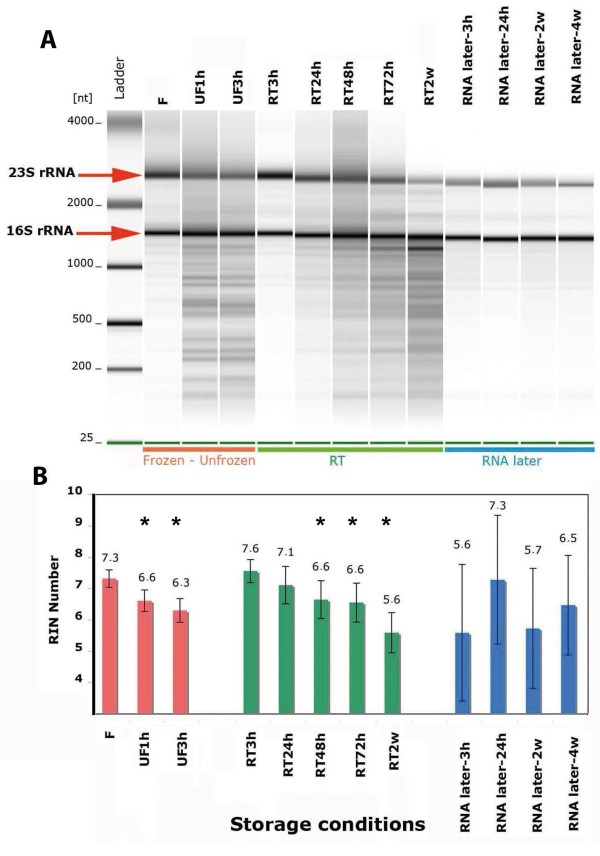
**RNA quality analysis.A**) Microcapillary electrophoresis patterns of total RNA extracted from fecal samples of one individual that underwent 12 different storage conditions: immediately frozen at −20°C (F); immediately frozen and then unfrozen (UF) during 1 h and 3 h; kept at room temperature (RT) during 3 h, 24 h, 48 h, 72 h and 2 weeks; mixed with commercial RNAse inhibitor solution (RNA later) and kept at room temperature during 3 h, 24 h, 2 weeks and 4 weeks. The equivalent to 1 mg of fecal material is loaded on each lane. A RNA fragment size (nt) marker was loaded in the first lane from the left side. **B**) Summary plot of average RNA integrity numbers (RIN) obtained with samples stored in the above 12 conditions. N = 11 individuals for the 88 samples stored without RNAse inhibitor. Standard deviation is indicated for each storage condition. N = 6 individuals for the 24 samples stored with RNAse inhibitor. Statistical analysis was performed using Poisson regression model (the star (*) means that the comparison with the frozen sample RIN number was significant with *p* < 0.05).

The integrity of total RNA is a critical parameter for metatranscriptomic analyses. Degradation of RNA compromises results of downstream applications, such as qRT-PCR [17] or microarray studies [18]. In order to assess the effect of storage conditions on total RNA recovery and integrity, we asked 11 volunteers (including the 4 above cited) to collect fecal samples and submit small aliquots to the following 8 conditions: immediately frozen at −20°C (F); immediately frozen and then unfrozen during 1 h and 3 h (UF1h, UF3h); kept at room temperature during 3 h, 24 h, 48 h, 72 h and 2 weeks (RT3h, RT24h, RT48h, RT72h, RT2w). The 88 samples so processed were brought at the laboratory and kept at −80°C until RNA was extracted and analyzed. Among these 11 volunteers, 6 individuals also agreed to provide fecal samples that after collection were immediately mixed with a commercial RNAse inhibitor solution (RNA later®) and kept at room temperature during 3 h, 24 h, 14 days and 1 month. The 24 samples obtained were brought at the laboratory at room temperature and directly processed for RNA extraction and analysis. RNA quality was examined by means of microcapillary electrophoresis (figure 3A shows the samples provided by one individual) and the average RNA integrity number (RIN) of all samples was compared for each storage condition (figure 3B). 

In all the conditions tested, the amount of RNA extracted was above 30 μg per 250 mg of stool, which is adequate for downstream analyses such as qRT-PCR and microarray experiments. When samples were immediately frozen after collection, extracted RNA had average RIN numbers above the value 7, which is the threshold acceptable for conducting metatranscriptomic studies [17,18].

However, unfreezing these samples during 1 h or 3 h before starting RNA extraction produced a strong RNA degradation, as illustrated in figure 1A by the fading of the 23S rRNA band and the appearance of numerous bands below the 16S rRNA. Decrease of the RIN numbers was significant after thawing samples for 1 h (*p* = 0.006, Wilcoxon paired test) and 3 h (*p* = 0.004, Wilcoxon paired test) compared to frozen samples. Conversely, when samples were kept at room temperature during few hours (3 h to 24 h) rather than immediately frozen after collection, total RNA extracted did not show signs of fragmentation and average RIN numbers were above 7. Longer storage periods at room temperature (more than 24 h) produced a progressive fragmentation of the RNA. Indeed, decrease in RIN number became significant when samples were kept at room temperature during 48 h (*p* = 0.036, Wilcoxon paired test). Finally, when samples were kept at room temperature in RNAse inhibitor solution, they showed less signs of fragmentation even after 4 weeks (figure 3A). In these conditions, however, there was a large RIN number variability among individuals (figure 1B).

Thus, our results indicate that the best storing condition to extract high quality RNA for metatranscriptomic analyses is to keep the stool samples at room (or low) temperature no more than few hours (< 24 h) after collection. Alternatively, samples can be kept at −20°C for longer periods as long as defrosting is prevented until the extraction of RNA starts in the laboratory. The RIN variability observed in samples mixed with RNA inhibitor could reflect an insufficient homogenization of hard stools (type 1 or 2 in the Bristol scale). Although the subjects could be asked to mix more thoroughly their stool after collection, this requirement is difficult to monitor. Therefore, the use of RNAse inhibitors may not be the best choice for semi or large-scale studies.

## Conclusions

Our study, although under a context of a small sampling size and other limiting parameters, suggests that storage conditions of stool samples can largely affect the integrity of extracted DNA and RNA and the composition of their microbial community. In light of our observations, our recommendation for semi or large-scale metagenomic and metatranscriptomic projects is to keep the samples at room temperature and to bring them in the laboratory within the initial 24 hours after collection. Alternatively, if bringing the samples during this period is not possible, samples should be stored immediately at −20°C in a home freezer. In this case, samples need to be transported afterwards in freezer packs to ensure that they do not defrost at any time. Mixing the samples with RNAse inhibitors and keeping them at home for longer periods of time (days) is not recommended since proper homogenization of the stool is difficult to monitor outside the laboratory.

## Methods

### Samples

Fecal samples were collected from healthy volunteers (n = 11), who did not receive antibiotics within the last three months. Samples were stored following 3 different procedures, which took into account volunteer’s compliance. In the first procedure, before being frozen at −80°C, each sample was kept at room temperature (RT) during different time periods (3 h, 24 h, 48 h, 72 h and 14 days). Time points before 3 h were not applicable, since volunteers needed this time to bring the samples from home to the laboratory. In the second protocol, samples were immediately frozen by the volunteers at their home freezer at −20°C and later were brought at the laboratory in a freezer pack, where they were immediately stored at −80°C. In order to test the effect of freezing and thawing episodes, some aliquots were defrosted during 1 h and 3 h before being stored at −80°C. In the third protocol, some volunteers agreed to collect their samples in tubes containing the RNAse inhibitor RNA Later® (Ambion) as indicated by the manufacturer instructions. The tubes were kept at room temperature during different time periods (3 h, 24 h, 14 days and 1 month) before RNA extraction. The protocol was approved by the Ethics Committee of the Vall d´Hebron University Hospital and all participants gave informed consent.

#### Assessing the quantity and quality of total RNA

For total RNA extraction, we modified the protocol described in Zoetendal et al. [15], which utilizes 15 g of fecal sample. Briefly, 200 mg of fecal sample were mixed with 500 μl TE buffer, 0.8 g Zirconia/silica Beads, 50 μl SDS 10% solution, 50 μl sodium acetate and 500 μl acid phenol. Physical disruption was conducted using a FastPrep apparatus. Following centrifugation of the lysate, nucleic acids were recovered from the aqueous phase and re-extracted with chloroform. DNA was selectively digested and the RNA was purified by using the RNeasy® mini kit (Qiagen) as described in the manufacturer instructions. A detailed protocol is provided in the supplementary information (See Additional file 3: Supplementary Methods).

An equivalent of 1 mg of each fecal sample was used for RNA quantification using a NanoDrop ND-1000 Spectrophotometer (Nucliber). The RNA was then examined by microcapillary electrophoresis using an Agilent 2100 Bioanalyzer with the RNA 6000 Nano Kit. The RNA quality was determined by the RNA integrity number (RIN), which is calculated from the relative height and area of the 16S and 23S RNA peaks and follows a numbering system from 1 to 10, being 1 the most degraded profile and 10 the most intact [14,19].

#### Assessing the quantity and quality of genomic DNA

Aliquots (250 mg) of each fecal sample were suspended in 0.1 M Tris (pH 7.5), 250 μl of 4 M guanidine thiocyanate and 40 μl of 10% N-lauroyl sarcosine. DNA extraction was conducted by mechanical disruption of the microbial cells with glass beads and recovery of nucleic acids from clear lysates by alcohol precipitation, as previously described in Godon et al. [20]. An equivalent of 1 mg of each fecal sample was used for DNA quantification using a NanoDrop ND-1000 Spectrophotometer (Nucliber). DNA integrity was examined by microcapillary electrophoresis using an Agilent 2100 Bioanalyzer with the DNA 12,000 kit, which resolves the distribution of double-stranded DNA fragments up to 17,000 bp in length.

#### Assessment of microbial composition through 16 S rRNA gene survey

In order to analyze bacterial composition, the V4 hypervariable region of the 16 S rRNA gene was amplified from the genomic DNA extracted from fecal samples by using two universal primers: V4F_517_17 (5’-GCCAGCAGCCGCGGTAA-3’) [21] and V4R_805_19 (5’-GACTACCAGGGTATCTAAT-3’) [22]. Multiplex identifiers (MIDs), which were used to perform tag pyrosequencing, were included upstream the forward primer sequence (V4F_517_17). PCR amplification was run in a Mastercycler gradient (Eppendorf) at 94°C for 2 min, followed by 35 cycles of 94°C for 30 sec, 56°C for 20 sec, 72°C for 40 sec, and a final cycle of 72°C for 7 min. PCR products were purified using PCR Purification kit (Qiagen, Spain) and subsequently sequenced on a 454 Life Sciences (Roche) Genome Sequencer FLX platform (UCTS, Hospital Vall d’Hebron, Barcelona, Spain).

Sequence analyses were performed using the Qiime pipeline [23]. Sequences were deposited in Genbank (Genbank: SRA055900). Uclust [24] was used to cluster sequences into OTUs (Operational Taxonomic Unit, taxa or species) at 97% sequence identity. Representative sequences for each OTU were aligned using PyNast against Silva 108 release database and taxonomy was assigned to the OTUs detected using blast and the Silva 108 release taxa mapping file. The results were summarized as the number of times an OTU was found in each sample and the taxonomic prediction for each OTU.

For beta diversity analysis we sub-sampled to 3080 sequences per sample to remove sequencing depth bias. A distance matrix was built based on weighted UniFrac method [25] and hierarchical cluster tree was built using UPGMA (unweighted pair group method with arithmetic mean).

### Statistic analyses

The Kolmogorov-Smirnov test was used to check the normality of data distribution. Comparisons of parametric normally distributed data were made by the Student’s test, paired tests for intra-group comparisons and unpaired tests for inter-group comparisons; otherwise the Wilcoxon signed rank test was used for paired data, and the Mann–Whitney *U* test for unpaired data. When dataset was small (n<5), we performed a Poisson regression model analysis using the function glm (Generalized Linear model) of R with the following formula [glm(formula = z ~ group + pair, family = poisson)]. This model is appropriate for modeling paired count data. *P* values < 0.05 were referred as significant.

## Authors' contributions

SC, MC, MG, CA carried out the sample collection and the molecular genetic studies, AE participated in the sequence and statistical analyses. JD, FA, FG participated in the design of the study. JR and CM participated in the design of the study, the interpretation of the results and the writing of the manuscript. All authors read and approved the final manuscript.

## Supplementary Material

Additional file 1 ** Table S1.** Detailed taxonomy assignment at the species level of the 24 samples. The taxonomy analysis is based on alignment performed using PyNast against Silva 108 release database and OTUs assignment using blast and the Silva 108 release taxa mapping file. Click here for file

Additional file 2 ** Figure S1.** Taxonomy analysis at the phylum level of the 24 samples based on alignment performed using PyNast against Silva 108 release database and OTUs assignment using blast and the Silva 108 release taxa mapping file. Click here for file

Additional file 3 ** Supplementary Methods. **Detailed description of extraction of total RNA from fecal samples. Click here for file
