# Supplementary figures and images for: Storage conditions of intestinal microbiota matter in metagenomic analysis
Source: BMC Microbiol. 2012 Jul 30;12:158. doi: 10.1186/1471-2180-12-158 (PMC3489833; doi:10.1186/1471-2180-12-158)

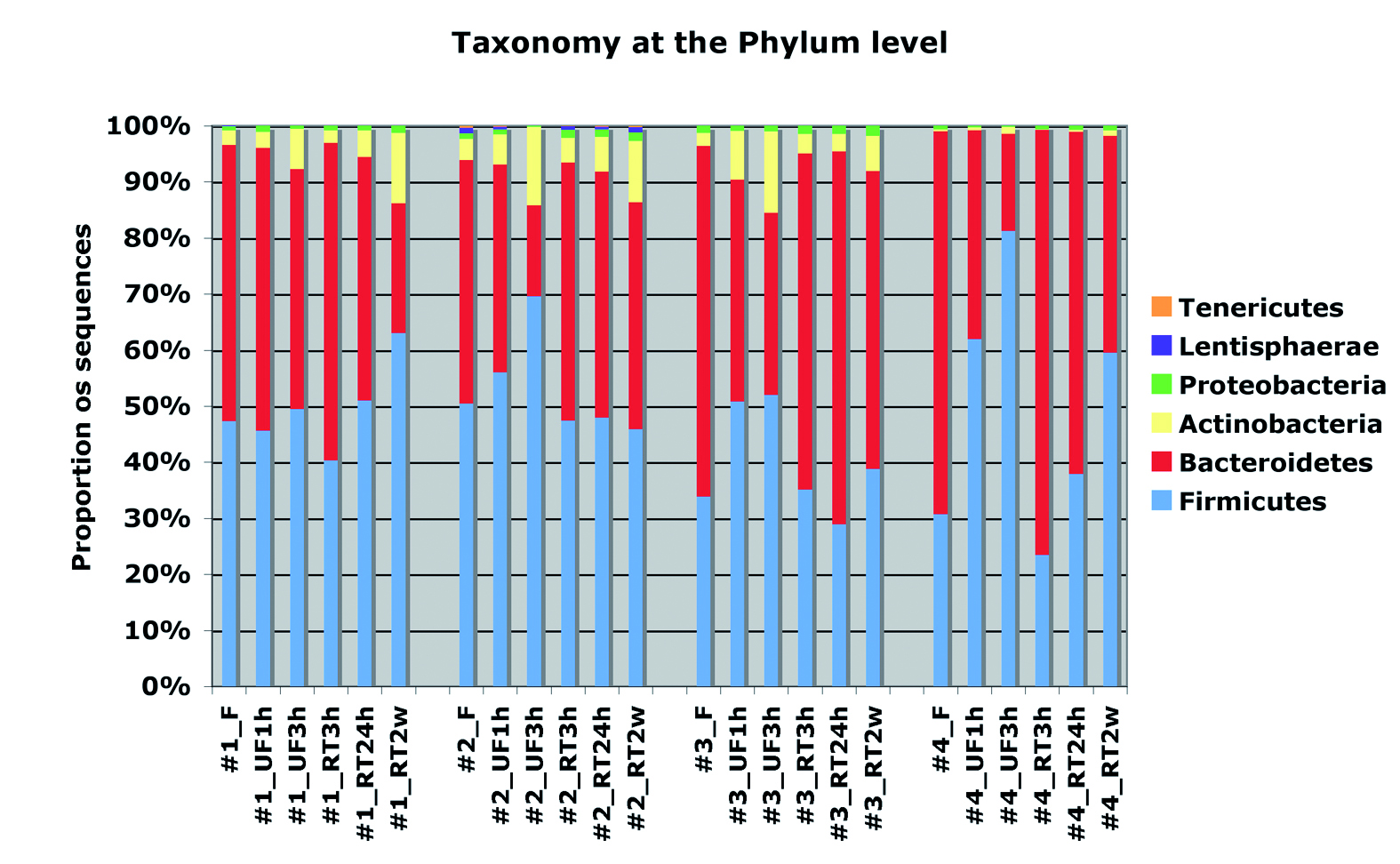

Supplement: Additional file 2 — Figure S1. Taxonomy analysis at the phylum level of the 24 samples based on alignment performed using PyNast against Silva 108 release database and OTUs assignment using blast and the Silva 108 release taxa mapping file. [file 1471-2180-12-158-S2.jpeg]
