# Supplementary material for: Storage conditions of intestinal microbiota matter in metagenomic analysis
Source: BMC Microbiol. 2012 Jul 30;12:158. doi: 10.1186/1471-2180-12-158 (PMC3489833; doi:10.1186/1471-2180-12-158)
Supplement: Additional file 3 — Supplementary Methods. Detailed description of extraction of total RNA from fecal samples. [file 1471-2180-12-158-S3.doc]

**Total RNA extraction protocol**

**Reagents**

- **RNase Away** (cat.no. 10328-011; Invitrogen)
- **Ice**
- **TE buffer**, pH 7.0 (cat. no. AM9861; Ambion)
- **Zirconia/silica Beads**, 0.1 mm (cat. no. 11079101z; Bio Spec Products Inc)
- **SDS** **10%**, dilute a 20% SDS solution (cat. no. AM9820; Ambion) to 10% SDS solution by using **DEPC-Water** (cat. no. 95284; Sigma).
- **Sodium Acetate** buffer, pH 5.2 ± 0.1 (cat. no. S7899; Sigma)
- **Chloroform:isoamylalcohol**, 24:1 (cat. no. 32715-5000; Bioblock) **CAUTION!** Chloroform:isoamylalcohol are harmful. Handle using appropriate safety equipment and measures.
- **RNeasy mini kit** (cat. no. 74104; Qiagen)
- **Ethanol** for molecular biology (cat. no. 1085430; Merck) **CAUTION!** Ethanol is flammable. Handle using appropriate safety equipment and measures.
- Stabilized **Phenol**, pH 4.1 (cat. no. GEXPHE01-0U; Eurobio) **CAUTION!** Phenol is toxic. Handle using appropriate safety equipment and measures.
- **DNase I** (cat. no. 10776785001; Roche)
- **10x Dnase-buffer**, make a solution consisting of 100 mM Tris-HCL, 25 mM MgCl_2_ and 5 mM CaCl_2_.
- **Glass Beads**, 2.5-3.5 mm (cat. no. 332124G; Merck)
- **RNALater** (cat. no. 7020; Ambion)

**Equipment**

- **Biosphere® Micro tube**, 1,5 ml (cat. no. 72206200; Sarstedth)
- **Biosphere® Micro tube**, 2.0 ml (cat. no. 72269500; Sarstedth)
- **Screw Tap Conical Micro tube**, 2.0 ml (cat. no. 602104; Alpha)
- **Tube**, 101x16.5 mm (cat. no. 80623; Sarstedth)
- **Eppendorf Centrifuge** (cat. no. 5415R; Eppendorf)
- **FastPrep® FP220A** (cat. no. 6001-220; MP Biomedicals)
- **RNA later collection tube?**

**Sample preparation**

Once samples arrived at the laboratory, 260-270 mg were aliquoted into 2.0 ml screw tap conical microtube with the help of sterile spatulas and frozen again at appropriate times before total RNA extraction.

For samples stored in RNALater, the volunteers are provided with tubes (101x16.5 mm) containing 2 ml of RNALater and 10 glass beads (2.5-3.5 mm) with their weight labeled in the tubes. The volunteers are asked to recover a measuring spoon of stool in each tube, shake vigorously and keep at room temperature. Once these tubes arrived to the laboratory they were weighed again and their concentration were corrected by adding RNLater in order to keep the same concentration than for the other storage conditions. At appropriate times the samples were processed as follows: once the concentration of the RNALater feces tubes with the glass beads were corrected by adding RNALater:

1. Transfer 1 ml of the suspension to 2.0 ml screw tap conical microtube
2. Centrifuge sample at 9,000g at 4 ºC for 5 min.
3. Remove and discard supernatant.

**Procedure for Total RNA extraction for all samples**

1. ALWAYS WORK ON ICE. Add into the sample tube 500 µl TE buffer, 0.8 g Zirconia/silica Beads, 50 µl SDS 10% solution, 50 µl sodium acetate and 500 µl acid phenol.

CAUTION! Phenol is toxic. Handle using appropriate safety equipment and measures.

1. Shake sample using the FastPrep:

- Speed of 5 m/s for 45 s.

- Cool on ice for 90 s.

- Speed of 5 m/s for 45 s.

1. Centrifuge sample at 13,400g in a microcentrifuge at 4 °C for 15 min.
2. Transfer 500 µl of the supernatant into a new 2.0 ml microtube.
3. Pipette 500 µl chloroform:isoamylalcohol (24:1).

CAUTION! chloroform:isoamylalcohol is harmful. Handle using appropriate safety equipment and measures.

1. Vortex for a few seconds.
2. Centrifuge sample at 14,000g in a microcentrifuge at 4°C for 7 min.
3. Transfer 150 µl of the aqueous phase into a 2.0 ml microtube. At this point treat the sample by using DNase digestion.

**DNA digestion using RNeasy mini kit**

1. Pipette 525 µl RLT buffer and mix by pipeting 5 times up and down.
2. Add 250 µl ethanol and mix by pipeting.
3. Apply sample in 500 µl proportions to RNeasy minicolumn, centrifuge at 9,300g in a microcentrifuge at 4 °C for 15 s, discard flow-through and repeat this until all portions are applied.
4. Replace the collection tube.
5. Pipette 350 µl RW1 buffer to column and centrifuge at 9,300g in a microcentrifuge at 4 °C for 15 s.
6. Discard flow-through.
7. Prepare a solution pipetting 10 µl DNase I solution to 8 µl 10X DNase buffer and 62 µl RNase free water for each sample plus one.
8. Mix gently
9. Pipette 80 µl of this solution to the middle of each column.
10. Incubate at room temperature for 20 min.
11. Pipette 350 µl RPE to column.
12. Centrifuge at 9,300g in a microcentrifuge at 4 °C for 15 s.
13. Discard flow-through.
14. Pipette 500 µl RPE to column.
15. Centrifuge at 9,300g in a microcentrifuge at 4 °C for 15 s.
16. Discard flow-through.
17. Pipette 500 µl RPE to column.
18. Centrifuge at 9,300g in a microcentrifuge at 4 °C for 2 min.
19. Discard flow-through.
20. Centrifuge at 9,300g in a microcentrifuge at 4 °C for 1 min.
21. Place column onto 1.5 ml micro tube.
22. Pipette 30 µl of RNase-free water onto column.
23. Incubate at room temperature for 1 min.
24. Centrifuge at 9,300g in a microcentrifuge at 4 °C for 1 min.
25. Pipette the 30 µl eluted onto column.
26. Incubate at room temperature for 1 min.
27. Centrifuge at 9,300g in a microcentrifuge at 4 °C for 1 min.
28. Samples can be stored at -80 °C.
